# Supplementary material for: Trauma‐informed co‐production: Collaborating and combining expertise to improve access to primary care with women with complex needs
Source: Health Expect. 2023 Jul 10;26(5):1895–914. doi: 10.1111/hex.13795 (PMC10485347; doi:10.1111/hex.13795)
Supplement: Supplementary file 1 — Supporting information. [file HEX-26--s001.docx]

**Interview topic guide for Bridging Gaps project participants**

1. Background information: How would you describe your ethnic background, gender, age range?
2. How did you first find out about the project and what made you want to get involved?
3. What have been the best things about the project? Why?
4. What have been the most difficult things about the project? Why? (Prompts: lockdown, not being able to meet face-to-face, changes in team)
5. What have been your concerns/ worries through the project (Prompts: confidentiality, issues of stigma, sharing experiences)? Did you ever feel frustrated by the project? Which bits and why?
6. What made it easier to get involved in the project? What made it harder?
7. What did you do when different people disagreed within the project? Could this have been managed differently?
8. Have you ever felt the GPs/researchers/others on the team took the project off in directions that you didn’t agree with? Which bits and why? How did you feel? Did you feel that you could challenge them?
9. Why do you think that some women have dropped out of the project? Could anything have been done differently about this?
10. Do you feel people listened to what you had to say? Can you give an example of when you felt listened to? Did you ever not feel listened to?
11. Did women in the group who are experts by experience/ service users support each other? How? Did you feel supported by other women within the group? How? Were there any difficulties with this?
12. Can you tell me what it was like to get involved in different aspects of the project and what you learnt?
    1. the storytelling and communication skills workshops
    2. sharing lived experiences
    3. going to GP surgeries and doing face to face workshops before Covid-19
    4. online trauma-informed care training
    5. online GP training and online GP meetings
    6. working together with GPs and researchers involved in the project?
13. Did you feel that everybody’s knowledge and skills were valued and included? Why/ why not?
14. How has the project made an impact on you? Other people? Has your view of GPs or GP practice staff (and researchers?) changed at all?
15. Do you think that the project has had any impacts on healthcare services?
16. What would you like the project to do in the future? In what way(s) would you like to be part of that?
17. Is there anything else you would like to add?

**Many thanks for your time.**

**Interview topic guide for Bridging Gaps staff partners**

1. Background information: How would you describe your ethnic background, gender, age range?
2. What made you want to get involved with the project?
3. What was your role within the project?
4. What have been the best things about the project? Why?
5. What have been the most difficult things about the project? Why? (Prompts: lockdown, not being able to meet face-to-face, changes in team)
6. What have been your concerns/ worries through the project? (Prompts: issues of stigma, sharing experiences)? Did you ever feel frustrated by the project? Which bits and why?
7. How did you share decision-making powers? When couldn’t you involve women in the decisions and why?
8. How was peer support encouraged? Where there any issues with this?
9. Why do you think that some women have dropped out of the project? Could anything have been done differently about this?
10. What did you do when different people disagreed within the project? Could this have been managed differently?
    1. Prompt: did you ever feel the researchers/ GPs/ others on the team took the project off in directions that the women didn’t agree with? Which bits? What happened?
11. Can you talk to me a bit about how confidentiality has been managed through the project?
12. What did you learn from the project? Did you learn anything particularly from (not all relevant for all collaborators – just say if not relevant to you):
    1. The Bridging Gaps women involved in the project?
    2. Working with researchers and project GPs?
    3. online trauma-informed care training
    4. the storytelling and communication skills workshops?
    5. Working with the GP practices?
13. Did you feel that everybody’s knowledge and skills were valued and included? Why/ why not? How did drawing on all different people's knowledge make a difference to how the project moved forward?
14. How has the project made an impact on you? Other people?
15. Do you think that the project has had any impacts on access to healthcare services?
16. If you were to do this project again with hindsight, what would you do differently?
17. What would you like the project to do in the future?
18. Is there anything else you would like to add?

**Many thanks for your time.**

**Interview topic guide for Bridging Gaps researchers involved in project operations**

1. Background information: How would you describe your ethnic background, Gender, Age range?
2. What made you want to get involved with the project?
3. What was your role within the project?
4. How did staff changes affect dynamics within the group (explore theme of trust)?
5. What have been the best things about the project? Why?
6. What have been the most difficult things about the project? Why? (prompt: lockdown, changes in team)
7. What have been your concerns/ worries through the project? Did you ever feel frustrated by the project? Which bits and why?
8. How did you share decision-making powers? When couldn’t you involve women in the decisions and why?
9. How was peer support encouraged? Where there any issues with this?
10. Why do you think that some women have dropped out of the project? Could anything have been done differently about this?
11. What did you do when different people disagreed within the project? Could this have been managed differently? (Prompt: did you ever feel the researchers/ GPs/ others on the team took bits of the project off in directions that the women didn’t agree with? Which bits? What happened?)
12. Can you talk to me a bit about how confidentiality has been managed through the project? (Prompt: Names/ faces at GP practice, issues of stigma)
13. What did you learn from the project? Prompts as needed: Did you learn anything particularly from:
    1. The Bridging Gaps women involved in the project?
    2. Academic GPs
    3. online trauma-informed care training
    4. the storytelling and communication skills workshops?
    5. Working with the GP practices?
14. Did you feel that everybody’s knowledge and skills were valued and included? Why/ why not?
15. How did drawing on all those different people's knowledge make a difference to how the project/ you moved forward?
16. How has the project made an impact on you? Other people?
17. Do you think that the project has had any impacts on access to healthcare services?
18. How does co-production need to change so that it can be more trauma-informed?
19. What would you like the project to do in the future?
20. Is there anything else you would like to add?

**Many thanks for your time**
